# Effects of different caffeine doses on fat oxidation and cardiovascular response during exercise at FATmax in overweight/obese female college students

**DOI:** 10.1080/15502783.2026.2670558

**Published:** 2026-05-12

**Authors:** Zhigang Gong, Shihong Yu, Zhen Lyu, Wenying Huang, Tianou Zhang

**Affiliations:** a Faculty of Physical Education, Jiangxi Normal University, Nanchang, Jiangxi, China; b Nanchang Yuzhang Middle School, Nanchang, Jiangxi, China; c Department of Health and Exercise Sciences, Laboratory of Exercise and Sports Nutrition, University of the Pacific, Stockton, CA, USA

**Keywords:** Caffeine, energy metabolism, aerobic exercise, dose–response, young women, blood pressure

## Abstract

**Purpose:**

This study examined the acute effects of different caffeine doses on fat oxidation and cardiovascular responses at rest and during exercise at the intensity of maximal fat oxidation (FATmax) in overweight/obese sedentary female college students.

**Methods:**

In a randomized trial, eleven participants (age: 20.2 ± 2.3 years; BMI: 26.36 ± 1.78 kg/m²) completed four conditions: placebo (cellulose) or caffeine at 3, 5, or 9 mg/kg. Each session comprised 60 min of seated rest followed by 40 min of treadmill walking at FATmax. Substrate oxidation (via indirect calorimetry), blood pressure, and the fingertip perfusion index (PI) were measured at specific time points: at rest (0, 30, and 60 min after capsule ingestion) and immediately post-exercise (100 min). Additionally, gas exchange and heart rate were recorded continuously throughout the entire 100-min session. Data were analyzed using SPSS 26.0 with two-way repeated-measures ANOVA (or Friedman test for non-normal data) and Bonferroni-adjusted post-hoc comparisons. Significance was set at *p* < 0.05.

**Results:**

Caffeine did not alter the resting heart rate or substrate oxidation. During exercise, caffeine at 5 and 9  mg/kg significantly increased the heart rate and blood pressure (*p* < 0.05), while caffeine at 3 mg/kg elicited no such cardiovascular effects. The PI decreased across all caffeine groups (*p* < 0.05). Caffeine at both 3 and 5 mg/kg enhanced fat oxidation compared to placebo and the 9 mg/kg dose (*p* < 0.05). Carbohydrate oxidation was lower with 5 mg/kg caffeine than with placebo (*p* < 0.05), and both the 3 and 5 mg/kg doses showed reduced carbohydrate oxidation relative to the 9 mg/kg dose (*p* < 0.05).

**Conclusion:**

Acute caffeine intake at 3 and 5 mg/kg enhanced fat oxidation during FATmax exercise in sedentary overweight/obese females, whereas 9 mg/kg provided no additional metabolic benefit. However, the 5 mg/kg dose was associated with increased cardiovascular strain, which was not observed with the 3 mg/kg. Therefore, a dose of 3 mg/kg appears to offer an optimal balance between stimulating fat oxidation and maintaining cardiovascular safety in this population during acute exercise.

## 1. Introduction

Caffeine (1,3,7-trimethylxanthine), a widely consumed alkaloid in coffee, tea, and energy drinks, is ingested daily by approximately 90% of Western adults at an average dose of 200 mg/day [1–3]. Caffeine's effects on exercise substrate metabolism remain a topic of debate, with empirical evidence reporting divergent outcomes – ranging from enhancement [4–7] to no significant effect [8–10]. This inconsistency may arise from exercise intensity-dependent metabolic regulation [11], wherein fat oxidation peaks at moderate intensities – a concept formalized as FATmax by Jeukendrup [12]. Exercising at FATmax represents the optimal strategy for maximizing fat utilization [13,14], thereby positioning it as a critical target for metabolic optimizing during exercise. Mechanistically, caffeine augments fat oxidation through adenosine A1 receptor antagonism, which elevates catecholamine levels (e.g. epinephrine) to stimulate hormone-sensitive lipase activity and accelerate adipose triglyceride lipolysis [15,16]. Simultaneously, phosphodiesterase inhibition increases intracellular cyclic AMP (cAMP), amplifying lipolytic signaling [17]. These processes collectively enhance plasma free fatty acid availability and skeletal muscle uptake during exercise [18,19], particularly at FATmax, where lipolytic flux aligns with metabolic demand.

Given the established potential of caffeine to enhance fat oxidation, its application during exercise at FATmax has garnered interest. A pivotal study by Ruiz-Moreno et al. [18] demonstrated that 3 mg/kg caffeine increased fat oxidation at FATmax in healthy, regularly exercising adults [19]. However, the efficacy and optimal dosing of this strategy remain unexplored in overweight/obese sedentary populations – a group with distinct physiological characteristics and a high practical need for metabolic optimization strategies [20,21]. Furthermore, no study has systematically compared multiple caffeine doses to define a dose‒response relationship for fat oxidation specifically at FATmax intensity.

To address these key questions, this study investigated the acute effects of three caffeine doses (3, 5, and 9 mg/kg) on fat oxidation and cardiovascular responses during FATmax exercise in sedentary, overweight/obese female college students. The aim was to identify a dose that optimally enhances fat oxidation while minimizing adverse effects, thereby providing targeted evidence for caffeine use in this group. While not a weight loss trial per se, elucidating these acute responses can inform the design of effective long-term strategies for optimizing metabolism in such a population.

## 2. Methods

### 2.1 Participants

Eleven overweight or obese female college students (age: 20.2 ± 2.3 years; body mass: 66.3 ± 3.7 kg; height: 158.7 ± 3.7  cm; BMI: 26.36 ± 1.78 kg/m²; body fat percentage: 37.76 ± 3.42%) volunteered to participate in this study. All participants were healthy Chinese females (i.e. non-smokers, with normal blood pressure and fasting glucose, and without known metabolic, cardiovascular, or chronic diseases) aged 18–24 years with a BMI greater than 24 kg/m² (classified as overweight or obese according to Chinese criteria [22] and a body fat percentage exceeding 30% [23]. Body mass (via an integrated scale) and body fat percentage (via bioelectrical impedance analysis, BIA) were assessed using a body composition analyzer. Measurements were taken in the morning after an overnight fast. These data were used to determine eligibility and to calculate individual caffeine doses (3, 5, 9 mg/kg based on body mass).

Additional inclusion criteria were as follows: (1) engaging in exercise fewer than three times per week and less than 500 MET-minutes per week of moderate-to-vigorous physical activity, as assessed by the International Physical Activity Questionnaire (IPAQ) over the preceding month; (2) no history of smoking within the past six months; (3) confirmed daily caffeine consumption of less than 70 mg on average, verified by a 7-day dietary recall prior to enrollment; (4) no caffeine allergy; (5) no use of any chronic prescription medications (including oral contraceptives), over-the-counter drugs, or dietary supplements (including vitamins) within the past month; (6) no history of cardiopulmonary diseases; and (7) not using oral contraceptives.

Written informed consent was obtained from all subjects and/or their legal guardian(s) after full disclosure of procedures and risks, with protocols approved by Jiangxi Normal University’s Ethics Committee (IRB-JXNU-PEC-2022001) in accordance with the Declaration of Helsinki.

### 2.2 Experimental design

A double-blind, placebo-controlled, randomized crossover design was employed in this study. Each participant underwent four experimental trials: one placebo trial and three caffeine trials with varying doses, with each trial separated by a one-week washout period and conducted on a weekly basis. To ensure blinding, the caffeine and placebo capsules were identical in appearance and taste, making them indistinguishable to both participants and researchers. The capsules were administered 60 min prior to the initiation of exercise. An independent researcher assigned alphanumeric codes to each trial to maintain blinding, and these codes were only revealed after the completion of data analysis. At the conclusion of the study, participants were informally asked about their perceptions of the treatment received in each session to gain qualitative insight into the blinding efficacy.

During each trial, participants ingested the assigned capsule and remained seated for 60 min. Following this rest period, they performed a 40-min running session at the FATmax intensity. Throughout the exercise, continuous measurements of carbohydrate and fat oxidation rates, as well as heart rate, were obtained using indirect calorimetry. All trials were conducted in a laboratory setting with controlled environmental conditions, including an ambient temperature of 21.4 ± 0.5 °C and a relative humidity of 53 ± 13%.

### 2.3 Pre-experimental trial

#### 2.3.1 General familiarization session

At least 1 week prior to any formal testing, all participants completed a general familiarization session. During this session, the participants were introduced to the laboratory environment, practiced walking on the treadmill while wearing the metabolic mask, and learned to use the Borg Rating of Perceived Exertion scale. This session aimed to minimize learning effects and anxiety during subsequent tests.

#### 2.3.2 Incremental exercise test for determining individual FATmax and VO_2_max

One week after the familiarization session, participants performed an incremental treadmill test to determine their individual FATmax (exercise intensity eliciting maximal fat oxidation) and VO_2_max (maximal oxygen uptake).

**Rationale for the test protocol:** To accurately characterize the relationship between exercise intensity and fat oxidation rate in sedentary, overweight/obese individuals, the protocol commenced at a low workload. The initial speed of 4.0 km/h was selected to cover the low-intensity range where FATmax is frequently observed in this population [24,25]. The test employed a continuous, graded increase in workload (via speed and incline adjustments) to guide participants to volitional exhaustion, thereby allowing for the concurrent assessment of maximal exercise capacity and substrate metabolism across a broad intensity spectrum.

**Description of the incremental protocol:** The formal test began directly with participants walking at 4.0 km/h at a 0% incline. After 2 min at this intensity, the speed was increased to 4.6 km/h, while the incline remained at 0% for a further 2 min. At the fifth minute, the speed was increased to 5.2 km/h, and the incline was raised to 1%. From the sixth minute onward, the incline was fixed at 2%, and the treadmill speed was increased by 0.6 km/h every minute until the participant reached volitional exhaustion. Exhaustion was defined as the inability to maintain the required speed despite strong verbal encouragement, which was provided throughout the test.

#### 2.3.3 Gas exchange data collection and analysis

Breath-by-breath gas exchange data (VO_2_ and VCO_2_) were collected continuously throughout the test using a metabolic cart (Metalyzer 3B, Cortex, Germany). For the determination of FATmax, the final 60 s of data from each distinct stage lasting at least 1 min (i.e. the periods at 4.0 km/h/0%, 4.6 km/h/0%, 5.2 km/h/1%, and each subsequent one-minute stage) were averaged. The fat oxidation rate (g/min) for each of these stages was calculated using the following stoichiometric equation: 1.695 × VO_2_ (L/min)–1.701 × VCO_2_ (L/min) [18,26]. FATmax was defined as the specific exercise intensity (corresponding to a unique combination of speed and incline) that elicited the highest calculated fat oxidation rate during the entire incremental test. In this study, the mean FATmax intensity corresponded to a walking speed of 4.78 ± 0.65 km/h at a 0% incline. The mean oxygen uptake at this intensity was 49.60 ± 2.80% of the participant's VO_2_max. VO_2_max was identified as the highest 30-second average VO_2_ value attained prior to exhaustion, with a group mean of 38.0 ± 5.7 mL/kg/min.

#### 2.3.4 Task-specific familiarization

Three days before the commencement of the main experimental trials, the participants completed a second, task-specific familiarization session. The purpose of this session was to allow participants to experience walking for approximately 10 min at their pre-determined, individual FATmax intensity (i.e. the specific speed and incline identified in section 2.3.3). This practice ensured participants' comfort, gait stability, and physiological adaptation to the target intensity before undertaking the prolonged 40-min experimental trials.

### 2.4 Standardizations

Upon meeting the inclusion/exclusion criteria, participants were instructed to abstain from all caffeine-containing products (e.g. coffee, tea, chocolate, and energy drinks) for the duration of the study. They were also asked to maintain their habitual physical activity patterns.

Dietary standardization. To control for potential dietary confounders, participants completed a detailed record of all food and beverages consumed in the 24-hour period prior to their first experimental trial. This was done under the researcher's guidance using a structured form that captured food types, brands, and portions. The participants were then provided with a copy of their own recorded diet and instructed to replicate it as precisely as possible in the 24 h preceding each of the subsequent three trials. Compliance with this replication was verbally confirmed at the beginning of each trial visit.

Estimated Nutritional Consistency. Based on the collected dietary records and the typical dietary patterns of young Chinese females with overweight or obesity, the average daily energy intake during this controlled period was estimated to be approximately 1600–2000 kcal. The macronutrient distribution was estimated at approximately 50%–60% from carbohydrates, 25%–30% from fat, and 15%–20% from protein. This estimated nutritional profile confirms that a consistent dietary baseline was effectively maintained across all experimental trials for each participant.

Additionally, they were required to avoid alcohol and obtain at least 8 h of sleep the night before each trial. All trials were scheduled to commence no earlier than 3 days after self-reported cessation of menses, with a fixed 7-day washout between sessions. While this approach does not strictly control for specific menstrual cycle phases, recent evidence suggests that substrate oxidation during moderate-intensity exercise [27,28] and the physiological response to acute caffeine intake during exercise [29,30] are not substantially different across the menstrual cycle. Therefore, our protocol represents a pragmatic and evidence-informed approach to standardizing testing conditions for this population.

### 2.5 Adverse events monitoring

To assess the safety and tolerability of the caffeine doses, adverse events were systematically monitored. Twenty-four hours after each experimental trial, the participants completed a standardized questionnaire. They were asked to report the presence and severity of symptoms commonly associated with caffeine intake, including headache, nervousness, gastrointestinal discomfort, palpitations, tremor, and sleep disturbance (specifically, difficulty initiating sleep on the night of the trial). The occurrence of any other unprompted adverse events was also recorded.

### 2.6 Experimental trials

On the afternoon of each experimental trial, participants arrived at the laboratory at 3:00 pm after fasting for at least 2 h. To minimize the potential effects of circadian rhythms on body temperature and other biological variables, each subsequent trial was scheduled exactly one week apart at the same time of day. Upon arrival, participants were given identical capsules containing either caffeine or a placebo. Both the participants and the experimenters were blinded to the capsule contents. Caffeine and placebo were packaged in identical capsules to ensure that the study remained double-blind. To ensure effective blinding across the three dose regimens, the total number of capsules administered per session was kept constant for each participant. The required caffeine dose was provided using active capsules, with the remainder of the fixed total made up by visually identical placebo capsules. The participants ingested their assigned capsule with 150 ml of water. They then remained seated for 60 min to allow for substance absorption.

Each trial consisted of a 100-minute session: 60 min of seated rest following capsule ingestion, followed by 40 min of treadmill exercise at the FATmax intensity. Caffeine was administered using commercially available tablets (Piping Rock Health Products, LLC., USA), while fiber tablets (Amway Nutrilite Inc., USA) served as the placebo. Dosing was based on body mass, with participants assigned to one of three regimens: 3 mg/kg, 5 mg/kg, or 9 mg/kg. The body mass used for dose calculation was measured during the first laboratory visit and remained fixed for all subsequent trials for that participant.

Throughout the trials, physiological data were continuously monitored using a gas analyzer system (Metalyzer 3B, Cortex, Germany) for breath-by-breath measurement of oxygen uptake (VO_2_) and carbon dioxide production (VCO_2_), along with a heart rate monitor (H10, Polar, Finland). VO_2_, VCO_2_, and heart rate were recorded continuously from capsule ingestion until the end of the 100-minute trial. For statistical analysis, the continuously acquired data were averaged over specific time intervals: VO_2_ and VCO_2_ data were averaged over consecutive 15-minute blocks during the rest period and over consecutive 5-minute blocks during the exercise period; heart rate data were averaged over consecutive 15-minute blocks at rest and over consecutive 5-minute blocks during exercise. Additional hemodynamic measures – including brachial blood pressure (Yuwell 666AR, China) and the fingertip perfusion index (Masimo Pronto-7, USA) – were recorded at 0, 30, 60, and 100 min (i.e. immediately upon completion of the 40-minute exercise bout).

Substrate oxidation rates were determined using established stoichiometric equations [18,26], which were applied to the time-averaged VO_2_ and VCO_2_ data from each respective block:

Fat oxidation (g/min) = 1.695 × VO_2_ (L/min)—1.701 × VCO_2_ (L/min).

Carbohydrate oxidation (g/min) = 4.585 × VCO_2_ (L/min)—3.226 × VO_2_ (L/min).

### 2.7 Statistical analysis

An a priori power analysis was conducted using G*Power 3.1.9.7. Based on the primary outcome of fat oxidation, a large effect size (*f* = 0.38) was anticipated owing to the wide dosing range and existing literature [29]. For a repeated-measures ANOVA (*α* = 0.05, power = 0.80, correlation = 0.5), this indicated a required sample size of *N* = 11. The final sample consisted of *N* = 11 eligible participants. A post-hoc analysis confirmed that with this sample, the achieved power to detect an effect size of *f* = 0.38 was 80.1%.

The study data were analyzed using SPSS 26.0 and presented as mean ±  standard deviation. Normality was assessed for all variables at each time point using the Shapiro‒Wilk test. The majority of data, including heart rate, blood pressure, and most time points for metabolic variables, were normally distributed and thus analyzed with Two-way Repeated Measures ANOVA (time × group). For these ANOVA models, the ‘time’ factor corresponded to the 15-minute (rest) and 10-minute (exercise) averaging blocks defined in Section 2.6. Sphericity was checked, with Greenhouse–Geisser correction applied when violated. For the few time points where the data violated normality (RER at 20 min exercise; CHO oxidation at 30 min exercise and 15 min rest; fat oxidation at 30 min rest and 40 min exercise), the non-parametric Friedman test was used for dose comparisons.

For significant ANOVA results, the main and interaction effects were examined. Significant interactions were followed by post-hoc tests comparing each caffeine dose to placebo. For significant main effects, Bonferroni-adjusted pairwise comparisons were performed. Effect sizes for significant outcomes were reported as partial eta-squared (ηp²), interpreted as small (≥0.01), medium (≥0.06), or large (≥0.14) [31]. The significance level was *p* < 0.05.

Owing to the double-blinded nature of the study, the exact administration sequence for each participant was not recorded post-randomization. Therefore, a formal statistical test for carryover or order effects could not be performed. However, the following design features were implemented to minimize such effects: (1) a fixed 7-day washout period between trials, which exceeds the typical elimination half-life of caffeine; and (2) complete randomization of the treatment order for each participant.

## 3. Results

### 3.1 Changes of heart rate with different doses of caffeine

**Figure 1.**
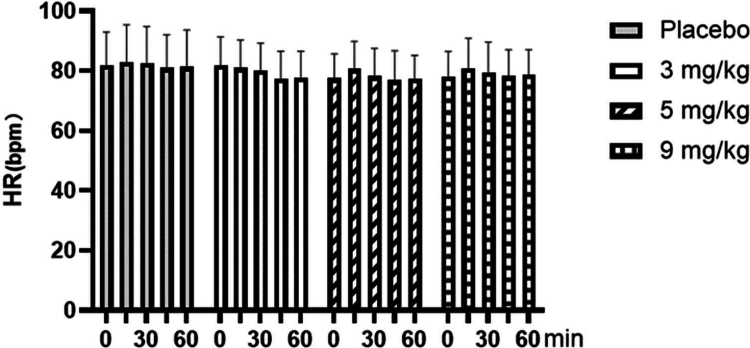
Changes of resting heart rate (HR) with different doses of caffeine.

As shown in Figure 1, for the resting heart rate, repeated-measures ANOVA showed no significant main effect of dose (F = 0.278, *p* = 0.841, ηp² = 0.023). Likewise, neither the main effect of time (F = 2.319, *p* = 0.060, ηp² = 0.061) nor the time × dose interaction (F = 0.797, *p* = 0.653, ηp² = 0.062) was significant.

**Figure 2.**
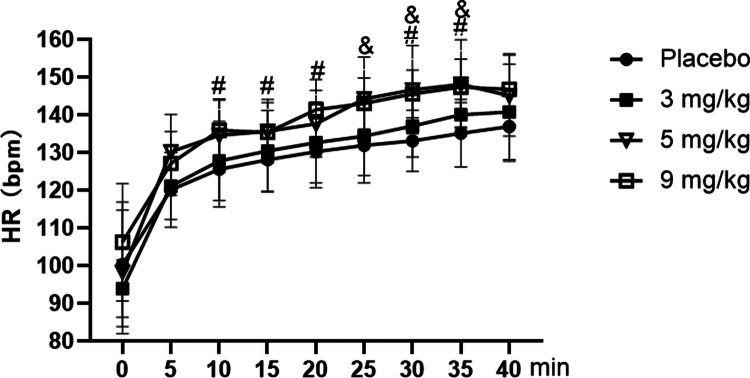
Changes in heart rate (HR) during exercise at FATmax with different doses of caffeine. Note: Compared to the Placebo group, # indicates a statistically significant difference in the 9 mg/kg caffeine group (*p* < 0.05), while & signifies a statistically significant difference in the 5 mg/kg caffeine group (*p* < 0.05).

As shown in Figure 2, for heart rate during exercise, repeated-measures ANOVA showed a significant main effect of dose (F = 4.373, *p* = 0.010, ηp² = 0.267). Post-hoc comparisons indicated that heart rate was highest with 9 mg/kg caffeine, followed by 5 mg/kg, with both being significantly higher than the placebo (*p* < 0.01). Caffeine at 3 mg/kg showed a non-significant increase compared to placebo (*p* > 0.05). The main effect of time was also significant (F = 185.468, *p* < 0.001, ηp² = 0.837), reflecting a progressive increase in heart rate throughout the exercise bout. Furthermore, the time × dose interaction was significant (F = 2.115, *p* = 0.035, ηp² = 0.078). Detailed time-point analysis revealed that, compared to placebo, 9  mg/kg caffeine had a significantly elevated heart rate at the 10th, 15th, 20th, 30th, and 35th minutes of exercise (*p* < 0.05), while 5  mg/kg caffeine showed a significant increase at the 25th, 30th, and 35th minutes (*p* < 0.05).

### 3.2 Changes of blood pressure with different doses of caffeine

**Figure 3.**
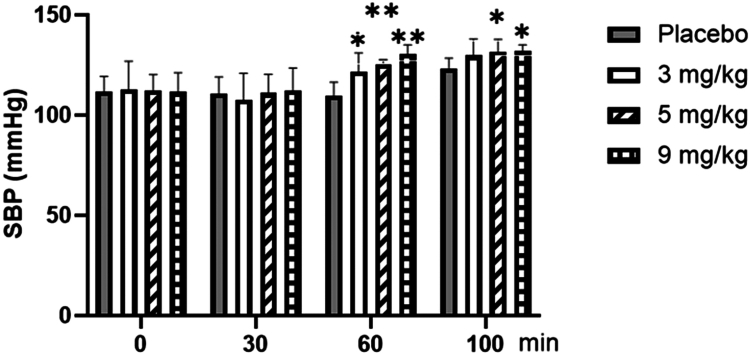
Changes in systolic blood pressure (SBP) with different doses of caffeine. Note: Compared with the Placebo group, * means *p* < 0.05; ** means *p* < 0.01.

As shown in Figure 3, for systolic blood pressure (SBP), repeated-measures ANOVA showed no significant main effect of dose (F = 0.2655, *p* = 0.063, ηp² = 0.181). However, the main effect of time was significant (F = 72.560, *p* < 0.001, ηp² = 0.668), with SBP at the 60th (rest) and 100th minute (post-exercise) being higher than at the 0th and 30th minutes (*p* < 0.01). Furthermore, the time × dose interaction was significant (F = 4.139, *p* < 0.01, ηp² = 0.256). Post-hoc analysis of this interaction revealed that at the 60th minute of rest, SBP was significantly higher with 3 mg/kg caffeine (*p* < 0.05), 5 mg/kg caffeine, and 9 mg/kg caffeine (both *p* < 0.01) compared to placebo. At the 100th minute (post-exercise), SBP remained significantly elevated with 5  mg/kg and 9  mg/kg caffeine compared to placebo (*p* < 0.05).

**Figure 4.**
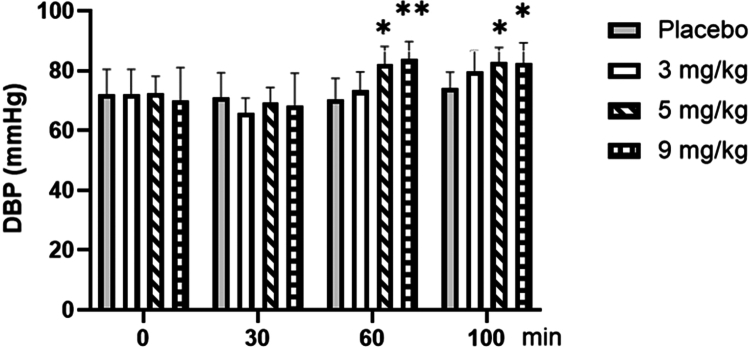
Changes in diastolic blood pressure (DBP) with different doses of caffeine. Note: Compared with the Placebo group, * means *p* < 0.05; ** means *p* < 0.01.

As shown in Figure 4, for diastolic blood pressure (DBP), repeated-measures ANOVA showed no significant main effect of dose (F = 1.808, *p* = 0.163, ηp² = 0.131). However, the main effect of time was significant (F = 40.248, *p* < 0.001, ηp² = 0.528), with DBP at the 60th (rest) and 100th minute (post-exercise) being higher than at the 0th and 30th minutes (*p* < 0.01). Furthermore, the time × dose interaction was significant (F = 5.344, *p* < 0.001, ηp² = 0.308). Post-hoc analysis of this interaction revealed that at both the 60th minute of rest and the 100th minute (post-exercise), DBP was significantly higher with 5 mg/kg caffeine (*p* < 0.05) and 9 mg/kg caffeine (*p* < 0.01) compared to placebo.

### 3.3 Changes of fingertip perfusion index (PI) with different doses of caffeine

**Figure 5.**
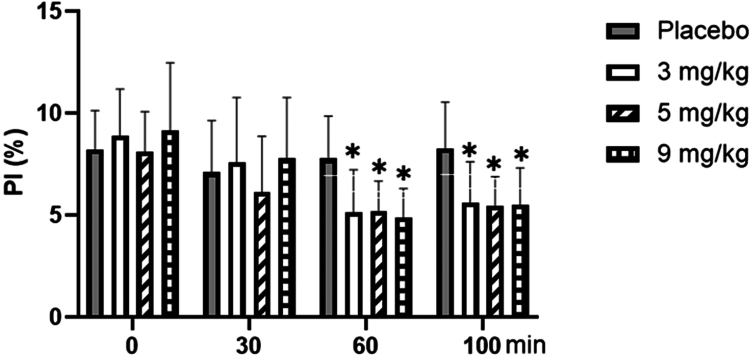
Changes in the fingertip perfusion index (PI) with different doses of caffeine. Note: Compared with the Placebo group, * means *p* < 0.05.

As shown in Figure 5, for the fingertip perfusion index (PI), repeated-measures ANOVA showed no significant main effect of dose (F = 1.051, *p* = 0.382, ηp² = 0.081). However, the main effect of time was significant (F = 30.256, *p* < 0.001, ηp² = 0.457). Post-hoc analysis for time revealed the following order (highest to lowest): PI at 0th minute >  30th minute ≈ 100th minute (post-exercise) >  60th minute. While PI increased significantly from the 60th to the 100th minute (*p* < 0.01), it remained significantly lower at the 100th minute compared to the baseline (0th minute) (*p* < 0.01). Furthermore, the time × dose interaction was significant (F = 7.261, *p* < 0.001, ηp² = 0.377). Simple effect analysis showed that at both the 60th and 100th minutes, the PI values were significantly lower in all three caffeine groups (3, 5, and 9  mg/kg) compared to placebo group (all *p* < 0.05).

### 3.4 Changes of fat oxidation with different doses of caffeine

**Figure 6.**
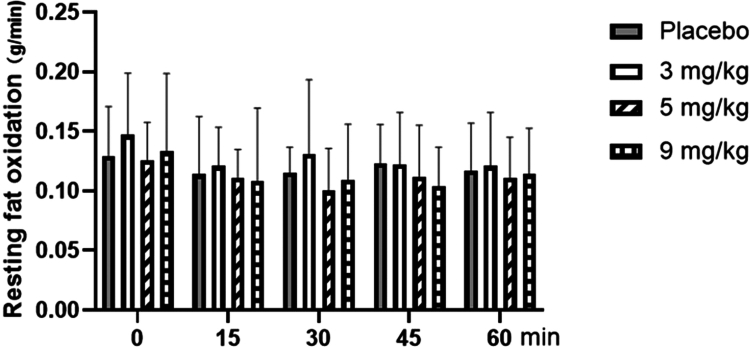
Changes in resting fat oxidation with different doses of caffeine.

As shown in Figure 6, for resting fat oxidation, repeated-measures analysis of variance showed that the main effect of the dose was not significant (F = 2.125, *P* = 0.114, ηp² = 0.150). The main effect of measurement time was not significant (F = 2.199, *P* = 0.072, ηp² = 0.058); the interaction between measurement time and dose was not significant (F = 1.013, *P* = 0.440, ηp² = 0.078).

**Figure 7.**
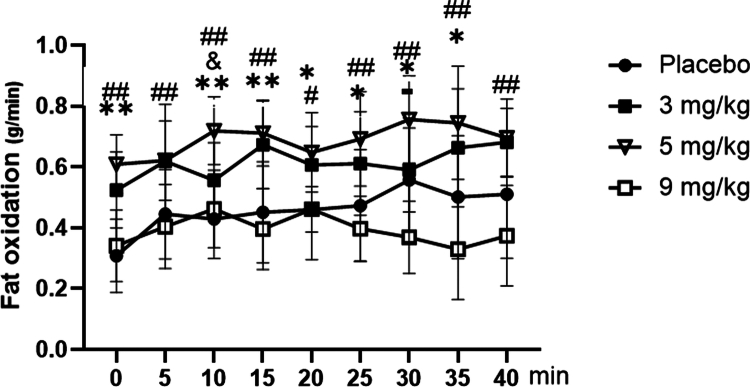
Dynamic changes in fat oxidation among groups during exercise. Note: Compared with the placebo group: *p* < 0.05 (*), *p* < 0.01 (**). Compared with the 9 mg/kg group: *p* < 0.05 (#), *p* < 0.01 (##). Between the 3 and 5 mg/kg groups: *p* < 0.05 (&). Summary of differences: The fat oxidation rates of the 3 and 5 mg/kg groups were significantly higher than those of the placebo group at most time points. The fat oxidation rate of the 5 mg/kg group was significantly higher than that of the 9 mg/kg group at most time points, with a significant difference also observed between the 3 and 5 mg/kg groups at time points such as 10 min.

**Figure 8.**
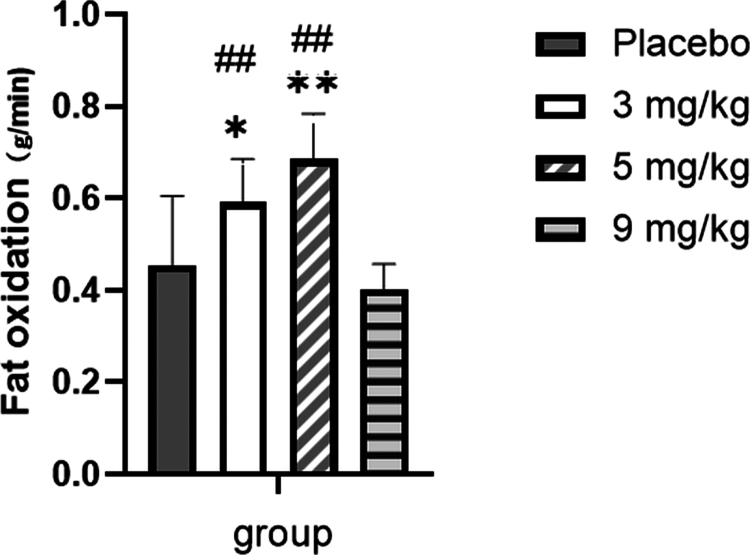
Comparison of total fat oxidation rates among groups during exercise. Note: Compared with the placebo group: *p* < 0.05 (*), *p* < 0.01 (**). Compared with the 9 mg/kg group: *p* < 0.05 (#), *p* < 0.01 (##).

As shown in Figure 7, for fat oxidation during exercise, repeated-measures ANOVA showed a significant main effect of dose (F = 6.837, *p* = 0.010, ηp² = 0.363), with oxidation rates generally increasing from 3 to 5 mg/kg caffeine but decreasing at 9 mg/kg. The main effect of time was also significant (F = 12.942, *p* < 0.001, ηp² = 0.519), indicating a progressive increase in fat oxidation throughout the exercise bout. Furthermore, the time × dose interaction was significant (F = 3.019, *p* < 0.001, ηp² = 0.201). Post-hoc analyses of dynamic fat oxidation during exercise revealed that the fat oxidation rates in the 3 mg/kg and 5 mg/kg groups were significantly higher than those in the placebo group at most time points (*p* < 0.05 or *p* < 0.01). Additionally, the fat oxidation rate in the 5 mg/kg group was markedly higher than that in the 9 mg/kg group at most time points (*p* < 0.01), and a significant intergroup difference was also detected between the 3 mg/kg and 5 mg/kg groups at 10  min (*p* < 0.05). For total fat oxidation over the course of exercise (Figure 8), compared with the placebo group, supplementation with 3 and 5 mg/kg caffeine resulted in a significant increase in total fat oxidation (*p* < 0.05 or *p* < 0.01), while no statistically significant difference was observed in the 9 mg/kg caffeine group (*p* > 0.05). Furthermore, total fat oxidation was significantly elevated in both the 3 mg/kg and 5 mg/kg caffeine groups relative to the 9 mg/kg caffeine group (*p* < 0.01).

### 3.5 Changes in carbohydrate oxidation with different doses of caffeine

**Figure 9.**
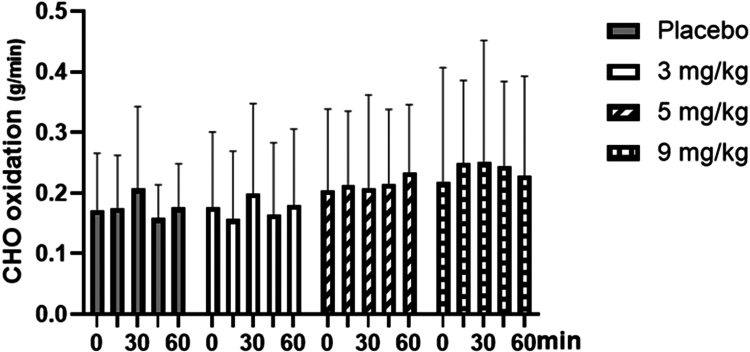
Changes in resting carbohydrate (CHO) oxidation with different doses of caffeine.

As shown in Figure 9, for resting carbohydrate oxidation, the repeated-measures ANOVA results showed that the main effect of the dose was not significant (F = 1.729, *p* = 0.178, ηp² = 0.126). The main effect of measurement time was not significant (F = 1.036, *p* = 0.391, ηp² = 0.028); the interaction between measurement time and dose was not significant (F = 1.422, *p* = 0.167, ηp² = 0.140).

**Figure 10.**
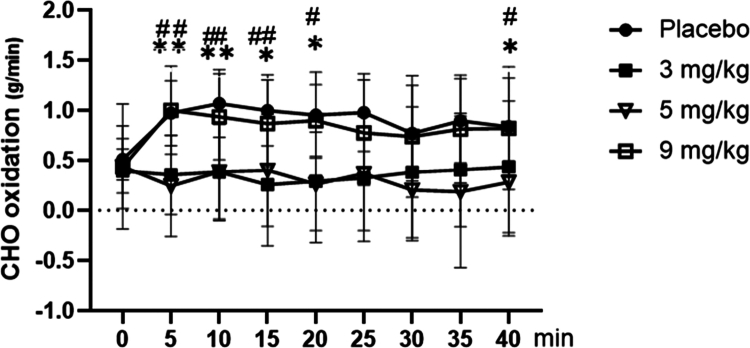
Dynamic changes in carbohydrate (CHO) oxidation among groups during exercise. Note: Compared with the placebo group: *p* < 0.05 (*), *p* < 0.01 (**). Compared with the 9 mg/kg group: *p* < 0.05 (#), *p* < 0.01 (##). Summary of differences: Both the 3 and 5 mg/kg groups exhibited significantly lower CHO oxidation rates than the placebo and 9 mg/kg groups at most time points (5–20 min, 40 min).

As shown in Figure 10, for carbohydrate (CHO) oxidation during exercise, repeated-measures ANOVA revealed a significant main effect of dose (F = 7.514, *p* < 0.001, ηp² = 0.385). Post-hoc analysis confirmed that CHO oxidation was significantly lower in the 5 mg/kg caffeine group compared to both the placebo and 9 mg/kg caffeine groups (*p* < 0.05 for both), whereas no significant differences were found between the 3 mg/kg caffeine group and either the placebo or 9 mg/kg caffeine group (*p* > 0.05). A significant main effect of time was also observed (F = 15.356, *p* < 0.001, ηp² = 0.809). CHO oxidation was highest at the onset of exercise and declined progressively throughout all subsequent time intervals (all *p* < 0.01 versus the initial value). The time × dose interaction was not significant (F = 1.565, *p* = 0.067, ηp² = 0.288).

Post-hoc analyses of dynamic changes in CHO oxidation during exercise (Figure 10) demonstrated that both the 3 mg/kg and 5 mg/kg caffeine groups exhibited significantly lower CHO oxidation rates than the placebo and 9 mg/kg caffeine groups at most time points (5–20 min, 40 min) (*p* < 0.05 or *p* < 0.01).

**Figure 11.**
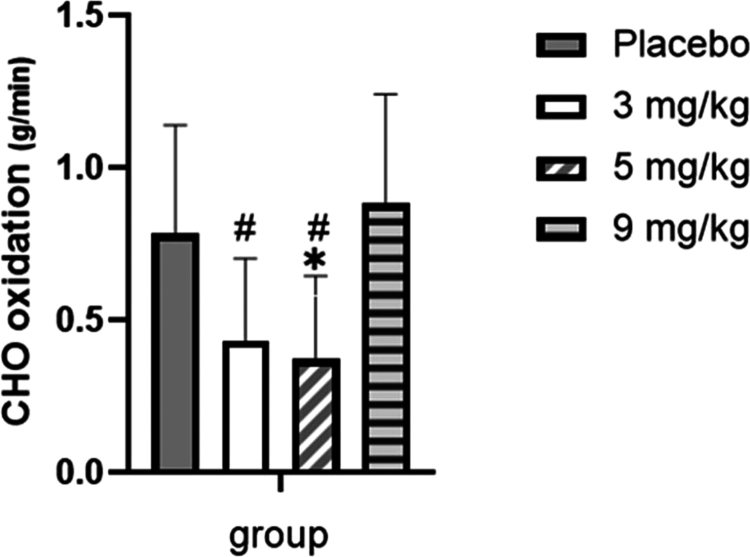
Comparison of total carbohydrate (CHO) oxidation among the groups during exercise.

For total CHO oxidation over the entire exercise period (Figure 11), compared with the placebo group, CHO oxidation decreased in both the 3 mg/kg and 5 mg/kg caffeine groups; specifically, the 3 mg/kg group showed a decreasing trend, but no statistically significant difference was observed (*p* > 0.05), while the 5 mg/kg group exhibited a significant decrease (*p* < 0.05), and no change was found in the 9 mg/kg group (*p* > 0.05). Furthermore, compared with the 9  mg/kg group, both the 3  mg/kg and 5  mg/kg groups showed a significant decrease in total CHO oxidation (*p* < 0.05).

### 3.6 Changes of respiratory exchange ratio(RER) with different doses of caffeine

**Figure 12.**
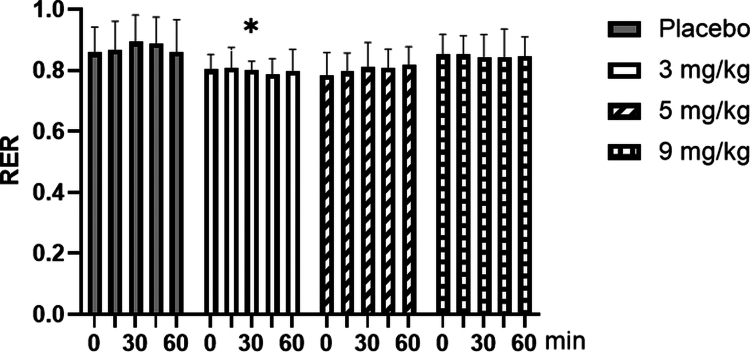
Changes in the resting respiratory exchange ratio (RER) with different doses of caffeine. Note: Compared with the Placebo group, * means *p* < 0.05.

As shown in Figure 12, for resting respiratory exchange ratio (RER), repeated-measures ANOVA showed a significant main effect of dose (F = 3.623, *p* = 0.022, ηp² = 0.232). Post-hoc comparisons revealed that 3  mg/kg caffeine had a significantly lower RER than placebo (*p* < 0.05). Neither the main effect of time (F = 0.385, *p* = 0.818, ηp² = 0.045) nor the time × dose interaction (F = 0.612, *p* = 0.828, ηp² = 0.065) was significant.

**Figure 13.**
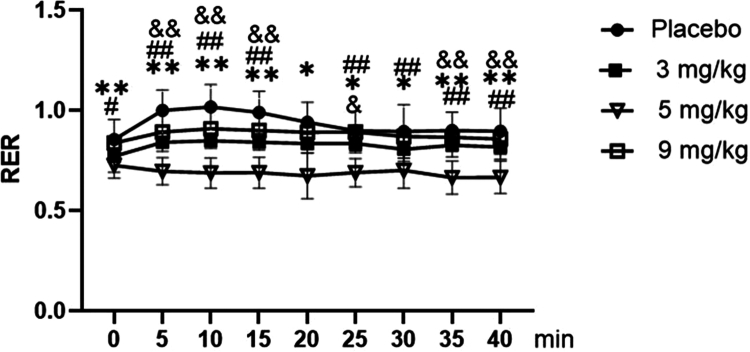
Dynamic changes in the respiratory exchange ratio (RER) among the groups during exercise at FATmax. Note: Compared with the placebo group: *p* < 0.05 (*), *p* < 0.01 (**). Compared with the 9 mg/kg group: *p* < 0.05 (#), *p* < 0.01 (##). Between the 3 mg/kg group and 5 mg/kg group: *p* < 0.05 (&). Summary of differences: The 5 mg/kg group exhibited significantly lower RER values than the placebo, 3 mg/kg, and 9 mg/kg groups at most time points from 0 to 40 min. The 3 mg/kg group also showed significantly different RER values compared to the placebo group at multiple time points (e.g. 5, 10, and 15 min).

As shown in Figure 13, for RER during FATmax exercise, repeated-measures ANOVA revealed a significant main effect of dose (F = 20.981, *p* < 0.001, ηp² = 0.636). Post-hoc analysis confirmed that RER values across groups followed this order (highest to lowest): Placebo > 9 mg/kg > 3 mg/kg > 5 mg/kg. A significant main effect of time was also observed (F = 3.690, *p* = 0.004, ηp² = 0.504). The RER values increased to a peak at 10 min and declined progressively, remaining slightly elevated at the end of exercise relative to baseline. The time × dose interaction was not significant (F = 1.479, *p* = 0.098, ηp² = 0.287).

Post-hoc analyses of dynamic RER during exercise (Figure 13) demonstrated that the 5 mg/kg group exhibited significantly lower RER values than the placebo, 3 mg/kg, and 9 mg/kg groups at most time points from 0 to 40 min (*p* < 0.05 or *p* < 0.01). The 3 mg/kg group also showed significantly lower RER values compared to the placebo group at multiple time points (e.g. 5, 10, and 15 min) (*p* < 0.05 or *p* < 0.01).

**Figure 14.**
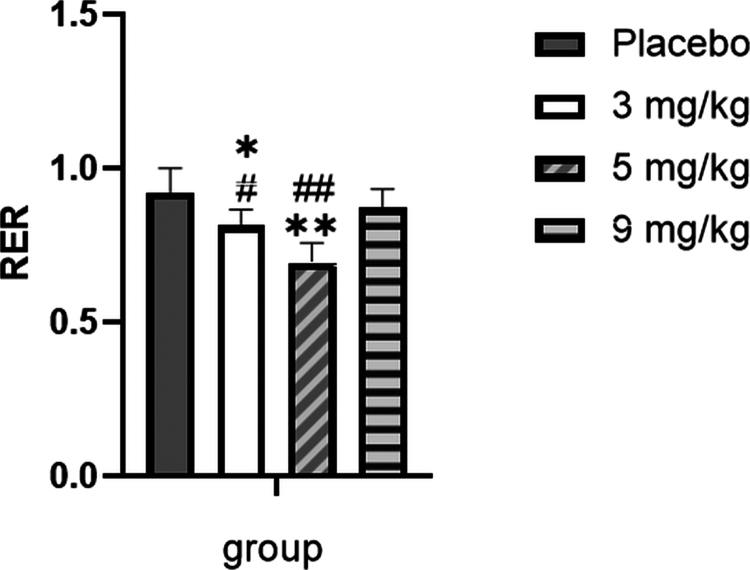
Comparison of total respiratory exchange ratio (RER) among the groups during exercise.

For total RER over the course of exercise (Figure 14), compared with the placebo group, supplementation with 3 and 5 mg/kg caffeine resulted in a significant decrease in total RER (*p* < 0.05 or *p* < 0.01), while no statistically significant difference was observed in the 9 mg/kg caffeine group (*p* > 0.05). Furthermore, the total RER was significantly lower in both the 3 mg/kg and 5 mg/kg caffeine groups relative to the 9 mg/kg caffeine group (*p* < 0.05 or *p* < 0.01).

## 4. Analysis and discussion

### 4.1 Effects of different doses of caffeine on fat oxidation during exercise at FATmax

The results of this study indicated that at rest, different doses of caffeine did not affect substrate metabolism (fat and carbohydrate oxidation) in overweight/obese sedentary female college students. It is worth noting that the mean VO2max of our participants (38.0 ± 5.7 mL/kg/min) was somewhat higher than the values typically reported in this population. This is likely explained by their young age (20.2 ± 2.3 years), the modest degree of overweight (mean BMI 26.4 kg/m²), and the fact that “sedentary” was defined as an absence of structured exercise rather than physiological incapacity. Thus, our sample represents a functionally capable subset of the overweight young adult female population, which should be considered when interpreting the generalizability of our findings. However, during exercise at FATmax, caffeine at 3 and 5  mg/kg significantly increased fat oxidation, while only the 5  mg/kg dose significantly decreased carbohydrate oxidation and RER.

The finding was consistent with those of previous studies. Numerous studies have consistently demonstrated that caffeine exerts a stimulatory effect on fat metabolism exclusively during exercise [11,14], while its impact on fat metabolism remains negligible during rest [17,18]. A recent meta-analysis by Conger et al. [32] found that fat metabolism was significantly increased after caffeine consumption [32]. The results showed that caffeine significantly increased the rate of fat oxidation and reduced the RER during exercise. The reason for this mechanism is that caffeine can stimulate the nervous system to secrete adrenaline, prompting fat cells to release free fatty acids into the bloodstream as a source of energy during exercise. At the same time, caffeine can also inhibit the activity of phosphodiesterase, maintain the level of cyclic AMP (cAMP) in cells, and further promote fat breakdown. This dual action is like the baton in a conductor's hand, skillfully adjusting the pace of fat burning within the body, causing it to peak during FATmax exercise.

Currently, there is a scarcity of studies investigating the impact of caffeine at 9 mg/kg on fat oxidation during exercise. This study represented the first attempt to examine the effects of this dose (9 mg/kg) on fat metabolism during exercise at FATmax using gas measurement techniques. The results from this experiment suggested that caffeine at 9 mg/kg did not demonstrate any noticeable influence on fat oxidation during exercise at FATmax; however, further investigation was warranted to elucidate the underlying mechanisms.

### 4.2 Effects of different doses of caffeine on the cardiovascular response during exercise at FATmax

This study demonstrated that caffeine had no significant impact on the resting heart rate. However, at the same relative external workload (FATmax intensity), caffeine at 3 mg/kg showed no effect on heart rate during exercise, whereas both caffeine at 5 and 9 mg/kg exhibited a noticeable increase. The finding was consistent with previous studies, such as Sung et al. report [33], which indicated that caffeine at 3  mg/kg consumption does not influence heart rate during intense exercise.

As expected, acute exercise at FATmax intensity itself elicited a significant increase in both systolic and diastolic blood pressure in the placebo condition (all *p* < 0.01), and significantly increased the fingertip perfusion index (PI) (*p* < 0.01), reflecting exercise-induced cutaneous vasodilation for heat dissipation. providing the physiological baseline against which the additional effects of caffeine were evaluated. In this study, alterations in arterial blood pressure and the fingertip perfusion index (PI) due to varying doses of caffeine were observed only after 60 min of intake. For example, blood pressure increased (only the 3 mg/kg dose affected systolic blood pressure), and the PI decreased, which was related to the absorption of caffeine in the body, and the blood caffeine content reached a peak 1 h after intake. Following exercise, both the 5 and 9 mg/kg doses exhibited a significant increase in arterial blood pressure, whereas no effect on blood pressure was observed with the 3 mg/kg dose; however, all three caffeine doses (3, 5, and 9 mg/kg) demonstrated a reduction in PI. These changes aligned with previous studies on blood pressure [33,34]. One hour after the intake of different doses of caffeine, the blood vessels of the fingertip microvascular bed contracted significantly, which can significantly reduce the blood flow to the fingers [35,36]. Previous studies [37] have demonstrated that caffeine induces transient contractions of arterial smooth muscle, as well as venous contractions, which were attributed to alterations in intracellular calcium concentrations. Caffeine exerted its effects on Ryanodine receptors in neurons, leading to the release of Ca^2+^ and subsequently enhancing muscle contraction force. Additionally, the influx of Ca^2+^ effectively prolonged the duration of muscle contraction. Meanwhile, caffeine also functions as a competitive inhibitor, binding to A1, A_2a_, and A_2b_ adenosine receptors [38]. Given that the activation of adenosine receptors led to vasodilation, acute caffeine consumption effectively inhibited A1 receptors, resulting in enhanced contraction. Therefore, all three caffeine doses (3, 5, and 9 mg/kg) can induce fingertip vasoconstriction because of their direct impact on smooth muscle cells and potentially through adenosine receptor inhibition.

There was a paucity of research investigating the correlation between caffeine consumption and PI. However, it was well-established that exercise led to blood redistribution, resulting in a significant increase in cardiac and muscular blood volume. The PI reflected peripheral blood distribution, with its decrease indicating reduced peripheral blood flow, augmented cardiovascular blood retention, and elevated lateral pressure on central blood vessels. Compared to placebo, each caffeine dose (3, 5, and 9 mg/kg) significantly decreased the PI during a 60-minute period of rest and post-exercise, thereby partially elucidating the mechanism underlying caffeine-induced elevation in blood pressure.

### 4.3 Reasonable caffeine intake during exercise at FATmax

Our evaluation of caffeine's metabolic effects revealed that acute intake did not significantly alter substrate utilization at rest. During exercise at FATmax intensity, however, caffeine at 3 and 5 mg/kg significantly increased fat oxidation while concurrently reducing carbohydrate oxidation. In contrast, the 9 mg/kg dose exhibited no discernible effect on substrate metabolism.

These results suggest a non-linear, likely inverted U-shaped, dose‒response relationship between caffeine and fat oxidation during FATmax exercise. The fat-oxidizing effect increased from 3 to 5 mg/kg but diminished at 9 mg/kg, which may exceed the optimal range and potentially trigger counter-regulatory responses. Thus, to maximize fat oxidation during exercise at FATmax, caffeine doses in the range of 3–5 mg/kg appear optimal, whereas a dose of 9 mg/kg provides no further benefit.

The cardiovascular assessment of caffeine revealed that it significantly increased blood pressure following complete absorption prior to exercise. Although this caffeine-induced elevation had minimal clinical relevance in the studied sample of healthy, active individuals, it could pose risks for those with pre-existing hypertension aiming to use caffeine for enhancing fat oxidation. Notably, one study indicated that the pressor effect of a daily 3 mg/kg caffeine dose diminished after 8 days of continuous use [39], suggesting the development of tolerance.

In the present study, caffeine at 3 mg/kg led to a slight increase in resting systolic blood pressure post-absorption, though both systolic and diastolic pressures remained unchanged immediately after exercise. In contrast, caffeine at 5 and 9  mg/kg resulted in significant increases in blood pressure, both at rest after absorption and immediately post-exercise.

Additionally, existing literature reports that caffeine intake can lead to adverse effects such as gastrointestinal discomfort, diuresis, and insomnia [39–41]. In the present study, systematic post-trial questionnaires were used to monitor these effects. Among the 11 participants, specific adverse events were reported only following the 9 mg/kg caffeine trial: one participant reported sleep disturbance (difficulty initiating sleep), and two different participants reported increased diuresis. No other consistent or notable side effects were documented across the placebo, 3 mg/kg, or 5 mg/kg trials.

While the pressor effect observed in this study was acute and transient, the literature suggests that some subjective side effects, such as nervousness or insomnia, may intensify with chronic use [42]. Given that the 9 mg/kg dose provided no additional fat-oxidation benefit but was associated with a higher likelihood of side effects, it cannot be recommended for this population.

Therefore, caffeine is recommended only for individuals seeking to improve fat oxidation during exercise who have no history of cardiovascular issues or heightened caffeine sensitivity. For overweight or obese female college students, a caffeine dose of 3 mg/kg during FATmax exercise is advisable as a safer option.

## 5 Conclusions and suggestions

This study demonstrates a clear dose-dependent effect of caffeine on substrate metabolism and cardiovascular responses in sedentary, overweight/obese women, contingent upon the activity state.

Key findings and interpretation: At rest, caffeine did not alter substrate oxidation but elicited a delayed pressor response. During FATmax exercise, however, caffeine at 3 and 5 mg/kg shifted fuel utilization towards increased fat oxidation and reduced carbohydrate reliance, aligning with potential metabolic benefits for weight management. Critically, caffeine at 9 mg/kg abolished this beneficial metabolic effect while concurrently producing the most pronounced cardiovascular stimulation (elevated heart rate and blood pressure) and incidence of side effects.

Practical implication and clear recommendation: Therefore, the classic “more is better” ergogenic strategy does not apply in this context. For the target population seeking to enhance fat utilization during moderate-intensity exercise, a caffeine dose of 3 mg/kg is recommended as an optimal strategy. This dose provides a significant metabolic benefit without imposing additional cardiovascular strain or increasing the risk of adverse effects, thereby offering a safer and more effective nutritional intervention approach.

Theoretical perspective: These results underscore the importance of considering both the dose‒response relationship and the exercise context when evaluating caffeine’s ergogenic potential, highlighting that its efficacy is profoundly state-dependent.

## 6. Limitations

Several limitations should be considered. First, owing to the double-blinded design, the exact administration sequence for each participant was not recorded post-randomization. Consequently, a formal statistical test for carryover or order effects could not be performed. However, the extended 7-day washout period (exceeding caffeine’s elimination half-life) and complete randomization of the treatment order make a systematic carryover effect unlikely. Second, the success of the double-blinding procedure was not formally evaluated using a quantitative index (e.g. Bang blinding index). This methodological choice was made because (a) the primary outcomes were objective physiological measures and (b) the pronounced physiological effects (e.g. increased heart rate) at higher caffeine doses could inherently compromise blinding, limiting the interpretability of such an index in this dose-ranging design. Third, while testing was scheduled at least 3 days post-menses, the fixed 7-day inter-trial interval meant that participants could have been in different menstrual cycle phases across trials. As ovarian hormones can influence substrate metabolism, this may have introduced variability into fat oxidation measurements. Our randomized crossover design ensures that this variability was evenly distributed across all conditions, making it unlikely to bias the primary dose–response comparisons, though it may have increased overall measurement noise. Finally, this study was specifically designed to investigate caffeine’s effect on substrate partitioning. Therefore, while total energy expenditure and ratings of perceived exertion (RPE) are valuable metrics, they were not analyzed as they fell outside the scope of the primary research hypothesis. Future studies may consider including these measures to provide a more comprehensive profile.
